# Regulation of polar auxin transport in grapevine fruitlets (*Vitis vinifera* L.) and the proposed role of auxin homeostasis during fruit abscission

**DOI:** 10.1186/s12870-016-0914-1

**Published:** 2016-10-28

**Authors:** Nathalie Kühn, Alejandra Serrano, Carlos Abello, Aníbal Arce, Carmen Espinoza, Satyanarayana Gouthu, Laurent Deluc, Patricio Arce-Johnson

**Affiliations:** 1 Departamento de Genética Molecular y Microbiología, Pontificia Universidad Católica de Chile, Alameda 340, PO Box 114-D, Santiago, Chile; 2 Department of Horticulture, Oregon State University, Corvallis, OR 97331 USA

**Keywords:** Auxin homeostasis, Fruitlet abscission, Grapevine, IAA, PIN efflux facilitators, Polar auxin transport

## Abstract

**Background:**

Indole-3-acetic acid (IAA), the most abundant auxin, is a growth promoter hormone involved in several developmental processes. Auxin homeostasis is very important to its function and this is achieved through the regulation of IAA biosynthesis, conjugation, degradation and transport. In grapevine, IAA plays an essential role during initial stages of berry development, since it delays fruitlet abscission by reducing the ethylene sensitivity in the abscission zone. For this reason, Continuous polar IAA transport to the pedicel is required. This kind of transport is controlled by IAA, which regulates its own movement by modifying the expression and localization of PIN-FORMED (PIN) auxin efflux facilitators that localize asymmetrically within the cell. On the other hand, the hormone gibberellin (GA) also activates the polar auxin transport by increasing PIN stability. In *Vitis vinifera,* fruitlet abscission occurs during the first two to three weeks after flowering. During this time, IAA and GA are present, however the role of these hormones in the control of polar auxin transport is unknown.

**Results:**

In this work, the use of radiolabeled IAA showed that auxin is basipetally transported during grapevine fruitlet abscission. This observation was further supported by immunolocalization of putative VvPIN proteins that display a basipetal distribution in pericarp cells. Polar auxin transport and transcripts of four putative *VvPIN* genes decreased in conjunction with increased abscission, and the inhibition of polar auxin transport resulted in fruit drop. GA_3_ and IAA treatments reduced polar auxin transport, but only GA_3_ treatment decreased *VvPIN* transcript abundance. When GA biosynthesis was blocked, IAA was capable to increase polar auxin transport, suggesting that its effect depends on GA content. Finally, we observed significant changes in the content of several IAA-related compounds during the abscission period.

**Conclusions:**

These results provide evidence that auxin homeostasis plays a central role during grapevine initial fruit development and that GA and IAA controls auxin homeostasis by reducing polar auxin transport.

**Electronic supplementary material:**

The online version of this article (doi:10.1186/s12870-016-0914-1) contains supplementary material, which is available to authorized users.

## Background

Auxins are a group of plant hormones involved in diverse developmental processes [1] through signaling cascades and transcriptional activation [2]. Among auxins, indole-3-acetic acid (IAA) is the most abundant and given that several processes finely tune its levels, this enables an optimized control of plant growth and development through its signaling [3].

The maintenance of IAA levels by biosynthesis, transport, degradation and conversion pathways is referred as auxin homeostasis [4]. *De novo* IAA biosynthesis maintains a steady supply of this hormone and occurs at specific sites, especially young tissues [5]. There are two major routes for IAA synthesis: the tryptophan (Trp)-dependent and Trp-independent pathways [3]. Trp-dependent biosynthesis of IAA is probably the main route occurring in plants, in which the two-step conversion of tryptophan to indole-3-pyruvic acid (IPyA) and then to IAA is the best understood pathway [6–8]. Indole-3-acetamide (IAM) is also a direct precursor of IAA [9], but the steps for IAM production in plants remain to be elucidated. The levels of IAA can also be modulated by conjugation (mainly to amino acids and sugars) and by degradation [10, 11]. Notably, IAA-Asp, IAA-Trp and IAA-Glu conjugation is irreversible, suggesting that these compounds are degraded through oxidation [12]. IAA-Trp conjugate is an IAA antagonist that counteracts IAA responses [13], increasing the IAA regulatory network complexity. Auxin inactivation is carried out by oxidation of IAA and IAA conjugates, giving rise to oxIAA, oxIAA-Asp and oxIAA-Glu, among others [14, 15]. Besides the metabolic control of IAA levels, its transport is crucial for regulating auxin homeostasis [16]. IAA movement from biosynthesis points to distant sites generates IAA gradients, which are crucial for its function [17, 18]. The directional movement of IAA is achieved by the asymmetrical arrangement of auxin efflux facilitators in the plasma membrane, called PIN-FORMED (PIN) proteins [19–21]. Together, all these mechanisms maintain optimal IAA levels, required for different developmental processes.

IAA plays important roles, especially during initial fruit development. IAA application in ovaries at anthesis triggers fruit set in the absence of pollination or fertilization, leading to the formation of parthenocarpic – seedless – fruits in Arabidopsis (*Arabidopsis thaliana*) and tomato (*Solanum lycopersicum*) [22, 23]. IAA injection into developing apple (*Malus x domestica*) fruits also produces an increase in fruit size and cell expansion [24]. Some evidence exists regarding the importance of auxin homeostasis in fruit growth and development. Treatments of unpollinated tomato ovaries with a polar auxin transport inhibitor leads to parthenocarpy. Correspondently, fruit formation is inhibited when pollinated ovaries are treated, correlating with higher IAA content [25]. This suggests that there is an optimal IAA concentration required for fruit set. Similarly, silencing of the tomato *SlPIN4* gene leads to the formation of parthenocarpic fruits [26]. Despite the reduction of *SlPIN4* expression should affect polar auxin transport, silenced lines maintain IAA levels similar to those of wild-type plants at anthesis, associated with increased IAA-Asp content prior to flowering, suggesting that some homeostatic mechanisms are able to mitigate IAA disruptions. It has been shown that IAA applications increase fruit size and reduces abscission in apple, while an excess of IAA results in reduced growth and fruit drop [24]. Altogether, these examples illustrate the importance of controlling auxin homeostasis for achieving normal fruit development.

Abscission is an important process that occurs during the initial development of fruits and determines fruit load, which in turn allows a proper distribution of assimilates from multiple sinks. This process is mainly controlled by the hormone ethylene [27, 28]. IAA is also involved in the control of fruitlet abscission, since it prevents the formation of the abscission zone (AZ) within the pedicel by decreasing ethylene sensitivity [29]. A constant IAA supply to the AZ comes from the developing fruit [25, 30] and application of polar auxin transport inhibitors results in abscission [31].

Despite the importance of polar auxin transport during the abscission process, our understanding about its regulation is limited. Changes in polar auxin transport and also in the expression of *PINs* genes during fruit growth have been reported [26, 30, 32] but signals underlying those changes remain unknown. IAA stimulates its own transport by inhibiting the endocytic step of PIN protein recycling [33] and by shaping actin filaments [34]. IAA also up-regulates the transcription of genes encoding PIN, increasing the PIN protein abundance [35–37]. Gibberellins (GAs) may also regulate the transport of auxins, by a positive regulation of polar auxin transport and induction of *PttPIN1* expression in the vascular cambium of hybrid aspen (*Populus tremula x tremuloides*) [38]. Furthermore, GAs also increase the abundance of PIN proteins in Arabidopsis [39]. Since GAs levels are high during initial fruit development in tomato and grapevine [23, 40, 41], they could have a role in the control of polar auxin transport during the abscission period.

Grapevine (*Vitis vinifera*) berries are non-climacteric fleshy fruits arranged in clusters formed by dozens of grapes [42]. During grapevine berry development, three phases can be distinguished according to the pericarp growth pattern. Phase I is characterized by an active berry growth; phase II corresponds to a lag phase, where no significant changes in berry size are observed; and phase III, is the period when growth resumes and ripening processes occur [43]. From flowering, phase I spreads over a period ranging from four to six weeks depending on the cultivar [44]. During this period, berry size increases mainly due to cell division and cell enlargement [45], and abscission process occurs [46] coincident with high ethylene content [47, 48]. Regarding IAA levels, there is some discrepancy about their variations during grapevine berry development. However, a decrease in IAA content from flowering to ripening has been reported [49], while IAA levels remain low and constant throughout berry development [50]. Nevertheless, no studies have reported neither the changes in IAA content during phase I nor the role of polar auxin transport and how these changes could be associated with the control of grapevine fruitlet abscission.

The importance of auxin homeostasis in grapevine fruits has been highlighted during berry ripening, when a decrease in IAA content was found to be correlated with an elevated IAA-Asp concentration; therefore, conjugation was proposed to enable ripening by reducing IAA content [49], as this hormone has been proposed to delay this process. However, there are no other examples of auxin homeostasis mechanisms controlling developmental processes in grapevine berries. In this work, abscission of grapevine fruitlets in relation to changes in polar auxin transport and transcript abundance of genes homologous to Arabidopsis *PINs* is studied. Since polar auxin transport is regulated by GA and IAA in model organisms [36, 38, 39] and both hormones are detected during phase I of grape berry development [40, 49, 51, 52], the role of these hormones in the regulation of polar auxin transport is also assessed. Finally, changes of IAA precursors, IAA conjugates and oxidation products are quantified during early stages of berry development. To our knowledge, this is the first report that evaluates hormonal regulation of polar auxin transport as well as changes in auxin-related compounds during initial berry development.

## Results

### Measurement of polar auxin transport in grapevine fruitlets

**Fig. 1:**
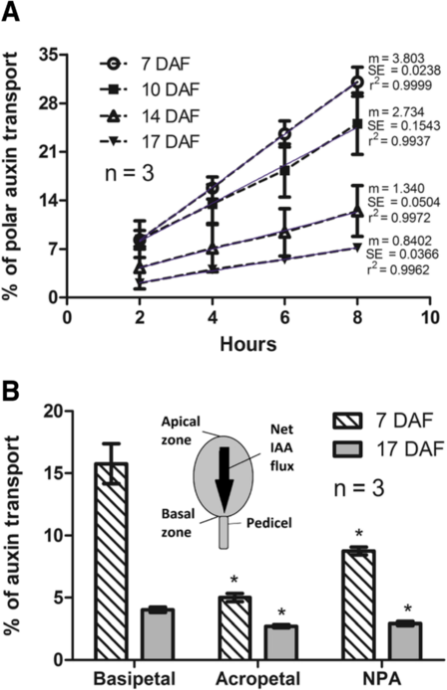
Basipetalauxin transport in grapevine fruitlets. **a** Percentage of auxin transport at 7, 10, 14, 17 days after flowering (DAF) after a 2-, 4-, 6- and 8-h transport period. Polar auxins transport was measured in excised fruits were percentage of auxin transport equals the percentage of radioactivity in receiver agars divided by the total radioactivity in the berries plus the receiver agars after a 8-h transport period. Linear regression is shown (blue line). m, slope; r^2^, coefficient of determination. **b** Percentage of auxin transport at 7 and 17 DAF after a 4-h transport period. Asterisk indicates that transport in acropetal and NPA controls is significantly different from basipetal transport (*p <* 0.05). Drawing represents fruitlet apical and basal zones, and IAA net flux direction. Error bars represent SE of three replicates

In order to determine if polar auxin transport occurs in grapevine fruitlets, a method for quantifying IAA movement across the berry was designed in excised fruits using radiolabeled IAA. The auxin transport rate in berries sampled between 7 and 17 days after flowering (DAF) was constant along the experiment duration (8 h) (Fig. 1a). Nevertheless, the slope of the linear regression decreased gradually from 7 to 17 DAF, indicating that the rate of auxin polar transport varies with the developmental stage. Next, an experiment was designed in order to compare basipetal (from the apical zone of the berry towards to the pedicel) and acropetal auxin transport (from the pedicel towards the apical zone of the berry) as well as the effect of NPA, an auxin transport inhibitor, on polar auxin transport (Fig. 1b). The amount of auxin effectively transported basipetally across the berries after 4 h of experiment was 15.8 % and 4.0 % at 7 and 17 DAF, respectively (Fig. 1b). Meanwhile acropetal transport (which is a measure of IAA diffusion), was 5.0 % and 2.7 % at 7 and 17 DAF, respectively. Net IAA flux, which was obtained by subtracting acropetal transport from basipetal transport after 4 h of experiment [53] was 10.8 % and 1.3 % at 7 and 17 DAF, respectively. IAA flux directionality was from the apical zone to the basal zone of the fruitlet. The IAA movement after the treatment with the auxin transport inhibitor, N-1-naphthylphthalamic acid (NPA), was assessed at 7 and 17 DAF. As shown in Fig. 1b, basipetal transport of IAA in NPA treated berries decreased from 15.8 % to 8.8 % and from 4.0 % to 2.9 % at 7 and 17 DAF, respectively. These results suggest that the rate of auxin transport varies with the developmental stage and that because at 7 DAF the auxin transport is decreased by NPA, possibly this is a polar transport.

### Effect of the polar auxin transport inhibitor NPA on grapevine fruitlet abscission

**Fig. 2:**
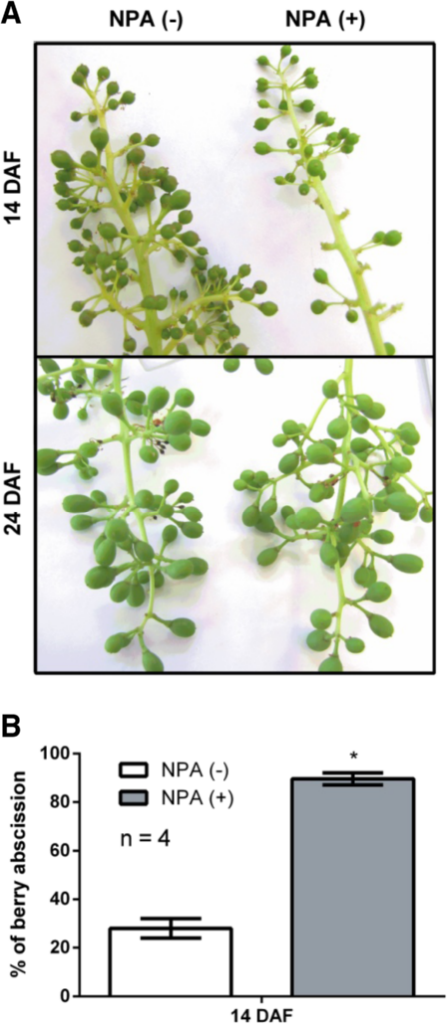
Effect of auxin transport inhibition on the abscission of grapevine fruitlets. **a** Representative image of 40 μM NPA-treated and control clusters. Treatment was performed at 10 and 20 DAF and visual inspection was done 4 days post treatment (DPT). **b** Estimation of fruitlet abscission in 14 DAF clusters showed in (**a**). Percentage of fruitlet abscission equals the percentage of 1 minus the ratio of berry number per cluster at 14 DAF and berry number in the same cluster at 10 DAF (see Additional file 3: Table S1). Error bars represent SE of four replicates (clusters). Asterisk indicates that fruitlet abscission rate in NPA-treated berries is significantly different from the corresponding value in control fruitlets (*p <* 0.05)

To determine if the inhibition of polar auxin transport has an effect on fruitlet abscission, 10 and 20 DAF fruitlets were treated with NPA and the effect was evaluated 4 days post treatment (DPT). As shown in Fig. 2a, NPA application in 10 DAF fruitlets produces abscission, leading to a remarkable reduction in fruit load at 14 DAF in comparison with control. However, NPA application in 20 DAF fruitlets had no evident effect on berry number at 24 DAF, when compared to control conditions. Abscission percentage of 10 DAF NPA-treated and control fruitlets was then quantified (Fig. 2b). It was found that NPA causes about 90 % of abscission, while control clusters have less than 30 % of abscission at 14 DAF. These results indicate that NPA treatment has a major effect on fruitlet abscission at 10 DAF, when the polar auxin transport seems to be higher.

### Abscission dynamics and polar auxin transport time course during grapevine fruitlet abscission

**Fig. 3:**
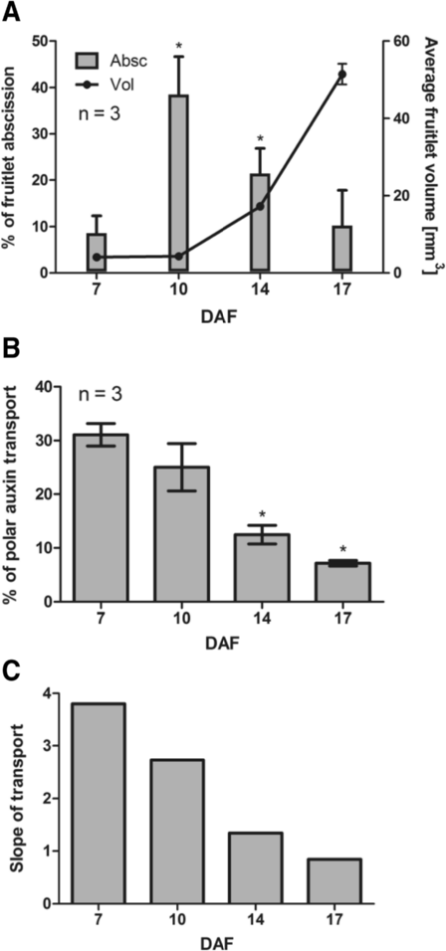
Abscission dynamics and time course of polar auxin transport in grapevine fruitlets (**a**) Estimation of fruitlet abscission at 7, 10, 14 and 17 DAF plotted with average fruitlet volume at the same DAF. Percentage of fruitlet abscission at 7, 10, 14 and 17 DAF equals the percentage of 1 minus the ratio of berry number per cluster at 7, 10, 14 and 17 DAF and berry number in the same cluster at 3, 6, 10 and 13 DAF, respectively (see Additional file 4: Table S2). **b** Percentage of polar auxin transport at 7, 10, 14 and 17 DAF after an 8-h transport period. For (**a**) and (**b**), asterisk indicates that fruitlet abscission or auxin transport is significantly different from the corresponding value at 7 DAF (*p <* 0.05). Error bars represent SE of three replicates. **c** Calculated slope of the 7, 10, 14 and 17 DAF regression lines presented in Fig. 1a

Initial development of grapevine fruitlets is characterized by a notorious fruit loss due to abscission, and depending on the cultivar it may occur rapidly within 10 DAF, or gradually, with some drop as late as 30 DAF [46]. In the present study, abscission in Autumn Royal cultivar was detected few days after flowering. The percentage of fruitlet abscission was determined comparing the berry number per cluster at 7, 10, 14 and 17 DAF relative to berry number in the same cluster at 3, 6, 10 and 13 DAF respectively. As shown in Fig. 3a, the percentage of berry abscission showed the highest values at 10 and 14 DAF, and then decreased at 17 DAF. The abrupt increase in berry abscission from 7 to 10 DAF precedes the berry volume increase that occurs from 14 DAF onwards (Fig. 3a). Interestingly, the increase in abscission from 7 to 14 DAF correlates with a decrease in the percentage of polar auxin transport in excised fruitlets (Fig. 3b) and with the slope of transport (Fig. 3c), which is a measure of the intensity of auxin transport, as stated in Shinkle et al. [54].

### Changes in transcript abundance of putative grapevine PIN genes during grapevine fruitlet abscission

**Fig. 4:**
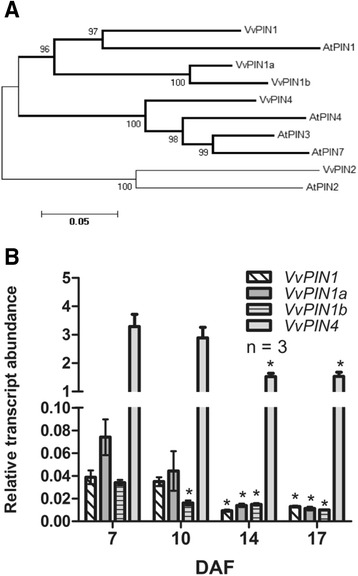
Phylogenetic tree of Arabidopsis (At) and putative grapevine (Vv) predicted PIN proteins and time course of *VvPINs* expression in grapevine fruitlets. (**a**) Neighbor-joining tree based on full-length protein alignment. Bootstraps of 1000 iterations are given. Scale bar shows the number of amino acid substitutions per site. Clades containing VvPINs whose transcripts were detected in grapevine fruitlets are highlighted with bold branches. **b** Relative transcript abundance of *VvPIN1*, *VvPIN1a*, *VvPIN1b* and *VvPIN4* was assessed in 7, 10, 14 and 17 DAF fruitlets. Transcript abundances are relative to the mean expression of the constitutive genes *VvUBI1* and *VvGPDH* (see Methods section). Error bars represent SE of three replicates. Asterisk indicates that transcript abundance is significantly different from the corresponding value at 7 DAF (*p <* 0.05)

In Arabidopsis, PIN family of auxin efflux facilitator proteins is composed of eight members, AtPIN1-AtPIN8 [16]. As only AtPIN1-AtPIN4 and AtPIN7 localize at the plasma membrane in a polar manner, correlating with the activity patterns of auxin-responsive reporters, they have been suggested to be responsible for polar auxin transport [20]. Hence, nucleotidic sequences of *AtPIN1*-*AtPIN4* and *AtPIN7* were used for a homology search in the Pinot Noir grapevine genome. This analysis allowed the identification of five gene models for putative grapevine *PIN* genes (*VvPINs*), called *VvPIN1*, *VvPIN1a*, *VvPIN1b*, *VvPIN2* and *VvPIN4.*To examine their fruit-specific expression, the presence of *VvPINs* transcripts in fruitlets and roots was assessed using RT-PCR. *VvPIN1*, *VvPIN1a*, *VvPIN1b* and *VvPIN4* were found to be expressed in developing berries and *VvPIN2* was found to be expressed only in roots (data not shown). Thus, only *VvPIN1*, *VvPIN1a*, *VvPIN1b* and *VvPIN4* where considered for further analyses. The predicted open reading frame of *VvPIN1*, *VvPIN1a*, *VvPIN1b* and *VvPIN4* encodes for 604, 555, 554 and 656 amino acid residues, respectively. AtPIN1 protein shares a 73 %, 61 % and 60 % identity with VvPIN1, VvPIN1a and VvPIN1b, while VvPIN4 shares a 76 %, 73 % and 74 % identity with AtPIN3, AtPIN4 and AtPIN7, respectively. The topology of the phylogenetic tree generated from the Arabidopsis and grapevine PIN amino acid sequences is shown in Fig. 4a. Next, relative transcript abundance of *VvPINs* was evaluated in fruitlets by qRT-PCR. Interestingly, transcript accumulation of all *VvPINs* showed their highest values at 7 DAF, and then a significant decrease is observed from 14 DAF onwards (Fig. 4b). This pattern correlates with the decrease in polar auxin transport, described previously (Fig. 3). Since *VvPIN4* showed the highest transcript abundance in comparison with the other *VvPINs* evaluated, it was chosen for immulocalization assays.

### Immunolocalization of putative VvPIN4 protein

**Fig. 5:**
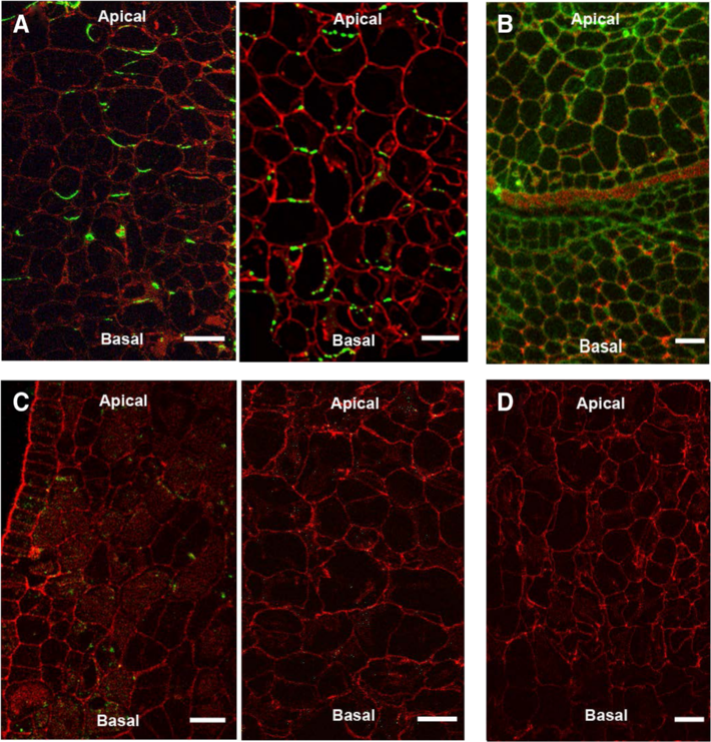
Immunolocalization of putative VvPIN4 protein on longitudinal sections of 10 DAF grapevine fruitlets. **a** Detection of putative VvPIN4 protein in pericarp cells using anti-AtPIN4. **b** Control with anti-Actin showing diffuse not polarized fluorescence. Background fluorescence observed on sections treated with anti-AtPIN4 (**c**) and anti-Actin (**d**) preimmune serum instead of antiserum. Two independent immunolocalization assays with anti-AtPIN4 and anti-AtPIN4 preimmune serum are shown. Red fluorescence is emitted by FM 4-64 membrane stain. Green fluorescence is emitted by secondary antibody conjugated to fluorescent dye. Bars = 30 μm

To determine whether high polar auxin transport and *VvPINs* transcript abundance registered at 10 DAF were consistent with the putative PIN localization at cellular level, immunolocalization using an antibody raised against Arabidopsis PIN4 was performed on grapevine fruitlets.. An *in silico* analysis shows that the putative VvPIN4 protein is predicted to be a membrane transporter (http://pfam.xfam.org/) and amino acid sequence alignment showed that the serine and threonine residues near the YPAPNP motif, whose phosphorylation is essential for PIN polarity [55], are present in VvPIN4 (data not shown). As shown in Fig. 5a, a clear polarized signal in the basal side of 10 DAF pericarp cells is observed when anti-AtPIN4 antibody was used. FM 4-64 membrane lipophilic dye was used to stain membranes indicates that the recognized proteins are membrane proteins. Control using anti-Actin shows diffuse fluorescence, indicating that polarized signal is indicative of VvPIN4 recognition (Fig. 5b).

### Effect of IAA, GA_3_ and IAA/GA_3_ treatments on polar auxin transport

We found a notorious increase in fruitlets abscission from 7 to 14 DAF that correlates with polar auxin transport and *VvPINs* transcript abundance decrease (Fig. 3 and Fig. 4b). Since IAA and GA regulate polar auxin transport in other model organisms [34, 38, 39], we wonder if the polar auxin transport might be regulated by IAA and GA in grape fruitlets as well.

We performed a search of cis-acting elements in *VvPINs* promoters, and multiple auxin- and GA-responsive elements in *VvPIN1*, *VvPIN1a*, *VvPIN1b* and *VvPIN4* promoter sequences were found (Additional file 1: Figure S1). Those elements were also identified in the promoter regions of Arabidopsis *PIN* genes [56–59].

**Fig. 6:**
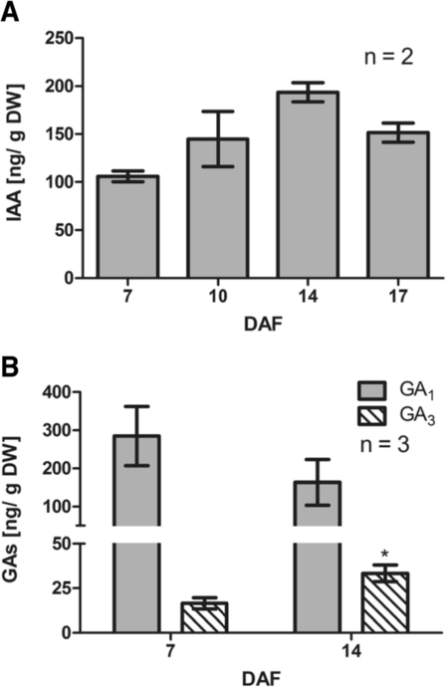
Endogenous GAs and free IAA content in grapevine fruitlets. IAA content at 7, 10, 14 and 17 DAF (**a**) and GA_1_ and GA_3_ content at 7 and 14 DAF (**b**) determined by LC-MS/MS. Asterisk indicates that concentration is significantly different from the corresponding value at 7 DAF (*p <* 0.05). Error bars represent SE of two (**a**) or three (**b**) replicates. DW, dry weight

**Fig. 7:**
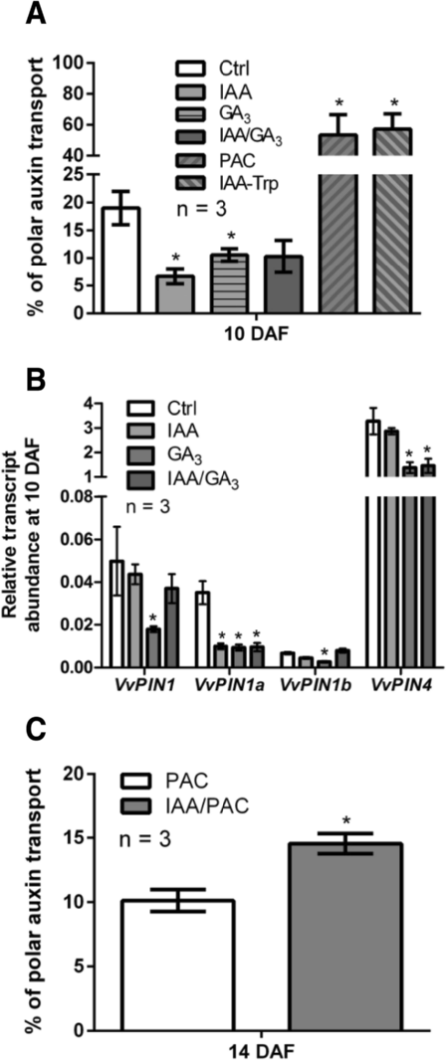
Effect of IAA and GA on polar auxin transport and *VvPINs* expression. Percentage of polar auxin transport after a 6-h transport period (**a**) and relative transcript abundance of *VvPIN1*, *VvPIN1a*, *VvPIN1b* and *VvPIN4* (**b**) in response to 1 μM IAA, 30 μM GA_3_, 1 μM IAA/30 μM GA_3_, 20 μM PAC and 1 μM IAA-Trp treatments at 10 DAF. Treatments were performed at 7 DAF and evaluation was done 3 DPT. Percentage of polar auxin transport after a 4-h transport period (**c**) in response to 20 μM PAC and 1 μM IAA/20 μM PAC treatments at 14 DAF. Treatments were performed at 12 DAF and evaluation was done 2 DPT. For (**a**) and (**b**), asterisk indicates that auxin transport or relative transcript abundance in treated fruitlets is significantly different from the corresponding value in control (Ctrl) berries (*p <* 0.05). For (**c**), asterisk indicates that polar auxin transport in IAA/PAC-treated berries is significantly different from the corresponding value in PAC-treated berries (*p <* 0.05). Error bars represent SE of three replicates

When endogenous amount of these hormones were quantified, free IAA levels were found to be within the range of 100-200 ng per gram of tissue, with no significant differences from 7 to 17 DAF (Fig. 6a). In the case of bioactive GAs, GA_1_ levels did not exhibit significant variations at the analyzed time points, while GA_3_ content increased significantly from 7 to 14 DAF (Fig. 6b). To test whether these hormones regulate polar auxin transport, IAA, GA_3_ and IAA/GA_3_ treatments were done at 7 DAF and the effect on polar auxin transport and *VvPINs* transcript abundance was evaluated 3 DPT. As shown in Fig. 7a, IAA, GA_3_ and IAA/GA_3_ treatments significantly reduced polar auxin transport. Interestingly, Paclobutrazol (PAC), an inhibitor of GA biosynthesis, and IAA-Trp, which exhibits an antagonist effects to IAA [13], caused an increase in polar auxin transport in comparison to both control and hormone treated samples (Fig. 7a). At the level of gene expression, GA_3_ treatment resulted in a decrease of the transcript abundance for all *VvPINs*, while IAA treatment reduced only *VvPIN1a* transcript abundance. The combined IAA/GA_3_ treatment showed a decrease in *VvPIN1a* and *VvPIN4* transcript abundance (Fig. 7b). As IAA positively regulates polar auxin transport through a positive feedback mechanism that alleviates elevated auxin levels [33, 34, 37], we hypothesized that the negative effect of IAA on polar auxin transport observed in our experiments (Fig. 7a) would be due to GA biosynthesis activation, since IAA induces GA oxidase genes [23, 60–63]. To test this, PAC and the combined PAC/IAA treatments were applied to 12 DAF berries,. The combined PAC/IAA treatment resulted in a significant increase in polar auxin transport compared with PAC treatment 2 DPT (Fig. 7c). It is possible to assume that in PAC/IAA treatment there is no induction of GA biosynthesis, and only IAA would account for any change in polar auxin transport. Taken together, these results show that GA and IAA exert a negative regulation over polar auxin transport and *VvPINs* expression during the abscission period of grapevine fruitlets. Yet, IAA can be a positive regulator of polar auxin transport when GA biosynthesis is inhibited.

### Measurement of IAA-related compounds during the abscission of grapevine fruitlets

**Fig. 8:**
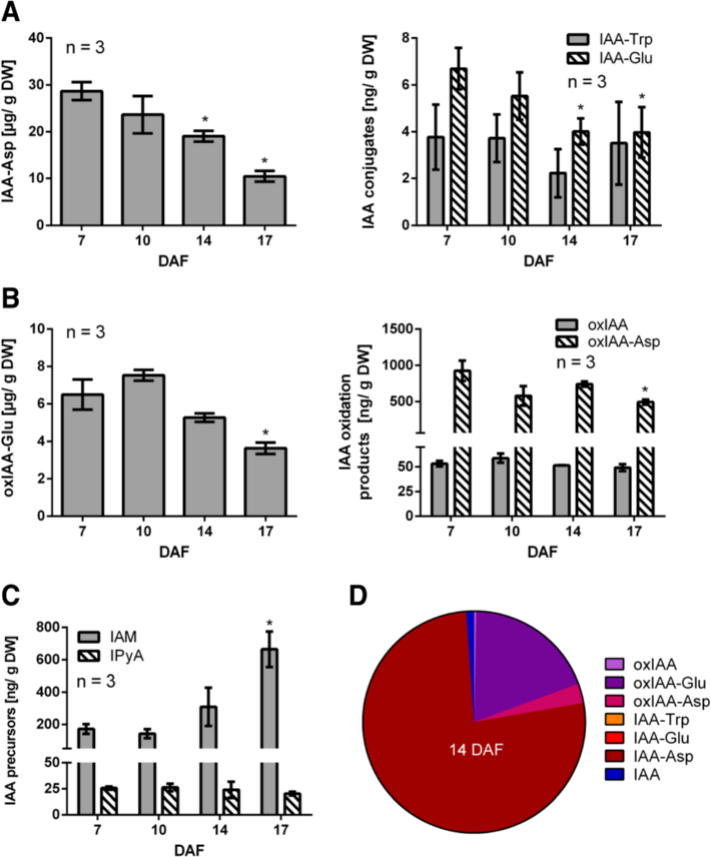
Content variation and relative abundance of endogenous IAA-related compounds in grapevine fruitlets. IAA conjugates (**a**), IAA oxidation products (**b**) and IAA precursors (**c**) content at 7, 10, 14 and 17 DAF determined by LC-MS/MS. Asterisk indicates that concentration is significantly different from the corresponding value at 7 DAF (*p <* 0.05). Error bars represent SE of three replicates. **d** Relative abundance of IAA-derived compounds at 14 DAF. Relative abundance equals the number of molecules per ng, estimated using molecular weight of each compound, divided by the total molecules. DW, dry weight

Since polar auxin transport steadily decreased during the abscission process (Fig. 3b, c), it would be expected a concomitant increase in IAA content at the end of the period, assuming that IAA biosynthesis is constant. However, IAA levels did not exhibit important variations, at least from 10 to 17 DAF (Fig. 6a). Therefore, it was hypothesized that other mechanisms could be involved in the control of IAA levels. In order to assess changes in IAA biosynthesis, conjugation and degradation, the levels of IAA precursors indoleacetamide (IAM) and indole-3-pyruvic acid (IPyA); IAA amino acid conjugates, IAA-Alanine (IAA-Ala), IAA-Aspartate (IAA-Asp), IAA-Tryptophan (IAA-Trp) and IAA-Glutamate (IAA-Glu); and IAA oxidation products, oxindole-3-acetic acid (oxIAA), oxindole-3-acetic acid-Glutamate (oxIAA-Glu) and oxindole-3-acetic acid-Aspartate (oxIAA-Asp), were analyzed by LC-MS/MS in grapevine fruitlets from 7 to 17 DAF (Fig. 8).

IAA-Asp was found to be the most abundant conjugated IAA form compared to IAA-Trp and IAA-Glu conjugates (Fig. 8a). On the other hand, IAA-Ala was not detected. It was also observed that IAA-Asp and IAA-Glu levels were significantly reduced from 7 to 14 DAF, while IAA-Trp showed no variations in the evaluated time points. When IAA-oxidation products were analyzed, it was found that the most abundant compound was oxIAA-Glu, while oxIAA-Asp and oxIAA-Glu were at lower levels (Fig. 8b). Also oxIAA-Glu as well as oxIAA-Asp decreased significantly at 17 DAF in relation to 7 DAF. Regarding IAA biosynthesis, the levels of IPyA precursor were constant, while IAM levels increased significantly from 7 to 17 DAF (Fig. 8c). The most abundant compounds derived from IAA were the irreversible IAA-Asp conjugate and the IAA-oxidation products, oxIAAGlu and oxIAA-Asp (Fig. 8d). These results, together with the observed changes in polar auxin transport, indicate that auxin homeostasis undergoes profound changes during a short developmental window in grapevine berries, when abscission process occurs.

## Discussion

### Auxin is basipetally transported in grapevine fruitlets

Directional flux of auxin underlies several developmental processes [17, 18]. In relation to fruit developement, basipetal transport in tomato fruitlets and sweet cherry pedicels has been already reported [25, 30].

In the present study, polar auxin transport was measured in grapevine fruitlets of Autumn Royal cultivar. Radiolabeled IAA applied to the apical zone of the berry was found to be basipetally transported, and the transport rate increased linearly during the period measured (Fig. 1a). In contrast, the basipetal transport was reported to plateau after 1.5 h in the pedicels of sweet cherry [30]. The reported stabilization could be due to a transport saturation caused by PIN protein delocalization in response to high levels of IAA, as shown by Vieten et al. [37].

At 7 DAF, about 16 % of the radiolabeled IAA taken up by the berry was transported into the basal zone after 4 h (Fig. 1b). Interestingly, basipetal transport was reduced by approximately 50 % after NPA treatment, indicating that measured IAA transport was polar. These values are similar to those obtained in excised hypocotyl sections of etiolated Arabidopsis and tomato seedlings after a 3-h transport period [53]. At 17 DAF, basipetal transport was lower, and NPA effect was not so marked. Acropetal transport reflects non-polar IAA movement, which includes passive diffusion and IAA movement mediated by non-polar PGP/MDR/ABCB efflux carriers [64–66] and the AUX/LAX family of auxin influx carriers [67–69]. At 7 DAF, acropetal transport was around one third of basipetal transport, which is higher than reported [30, 53]. This could be explained by an increased abundance of non-polar auxin transporters. At 17 DAF acropetal transport was lower compared with 7 DAF, showing that non-polar IAA movement also changes with berry age.

Basipetal VvPIN distribution supports basipetal auxin transport determined using radiolabeled IAA. VvPIN4 putative protein was localized in the basal side of pericarp cells at 10 DAF when anti-AtPIN4 antibody was used (Fig. 5). Even though we do not have enough evidence to state that AtPIN4 only recognizes VvPIN4 and not the other VvPINs, the polarized signal observed at the basal side of the cells strongly suggests that grapevine PIN auxin efflux facilitators are recognized by this antibody.

### Inhibition of auxin transport causes abscission in grapevine fruitlets

Fruitlet abscission is a morphogenetic process that depends on many factors. Among endogenous factors, hormones play a crucial role. Ethylene is the main hormone responsible for fruit abscission [27, 28], and a fine-tuning of the abscission process is a result of ethylene sensitivity modulation, which is known to depend on polar auxin transport [29].

Inhibition of polar auxin transport by NPA increased fruitlet abscission at 10 DAF (Fig. 2), indicating that polar auxin transport maintenance contributes to fruit retention. Notably, same treatment had no effect at 20 DAF. It has been previously reported that application of NPA to apple pedicels at post-bloom stage increases fruit abscission [70]. Nevertheless, to our knowledge differential effect of NPA depending on the developmental stage has not been investigated. It is possible that NPA treatment at 20 DAF has no effect on fruit load because berry abscission process has already ended at this time. In the same line, ethylene content is lower at 17 DAF compared to previous days (Additional file 1: Figure S2, Additional file 2). Thus, modulation of ethylene sensitivity by polar auxin transport is probably no longer required at this time.

### Abscission increase correlates with a decrease in polar auxin transport and transcript abundance of putative grapevine PIN genes

Abscission increases significantly from 7 to 14 DAF, preceding the sharp increase in berry size occurring from 14 DAF onwards (Fig. 3a). It is possible to suggest that the plant ensures fruit retention before promoting fruit growth, in order to avoid futile destination of resources into tissues that may abscise. Abscission increase could be the result of reduced amount of transported IAA and/or lower transport intensity (Fig. 3b and c). Similar results were obtained in sweet cherry, where transport intensity decreased prior to fruit abscission [31]. Polar auxin transport decrease was not so marked as abscission increase from 7 to 10 DAF, but we propose that slight changes in auxin homeostasis are enough to control developmental processes, such as abscission. Under the experimental conditions assayed, it was not possible to measure polar auxin transport before 7 DAF, but one would expect it to be even higher, as the highest values of auxin transport intensity are registered as early as three days from anthesis through sweet cherry pedicels, during cell division phase [30].

Reduction in polar auxin transport correlates with a decrease in *VvPINs* transcript abundance from 7 to 17 DAF (Fig. 4b). Mounet et al. [26] also reported a reduction of tomato *PIN* expression during fruit development, with the highest levels at anthesis and four days post-anthesis for all the five *SlPINs*. Changes in *VvPIN* transcripts might contribute to the observed decrease in polar auxin transport, although changes in protein abundance and localization could also be involved.

### Polar auxin transport is regulated by IAA and GA

Auxin can modify its own transport by up-regulating PIN transcription, as shown in Arabidopsis [36, 37]. Also, GA activates polar auxin transport, as reported in hybrid aspen and Arabidopsis [38, 39]. So, it was proposed that IAA and GA could be involved in the regulation of polar auxin transport, as both hormones are detected in grapevine fruitlets.

As shown in Fig. 3, polar auxin transport decreases during grapevine fruitlet abscission, thus if a positive regulation of IAA and GA over this transport occurs as reported in Arabidopsis and hybrid aspen, their levels should decrease accordingly. However, IAA and active GA_S_ did not present the expected pattern (Fig. 6) and possibly these hormones do not act in grapevine as previously reported. In fact, inhibition of polar auxin transport by IAA and GA_3_ was not expected (Fig. 7a), despite it was consistent with its activation after PAC and IAA-Trp treatments. At molecular level, *VvPINs* were all down-regulated by GA_3,_ while only *VvPIN1a* transcript abundance was affected by IAA (Fig. 7b). We hypothesized that the IAA effect on polar auxin transport was through GA biosynthesis activation. As expected, when GA biosynthesis was blocked with PAC, IAA was able to activate auxin transport (Fig. 7c).

If IAA induces GA biosynthesis, then IAA and the combined IAA/GA_3_ treatments should result in *VvPIN* down-regulation, but this was true only for *VvPIN1a*. Perhaps there is a balance between the putative inducing role of IAA on *VvPINs* expression and its presumed ability to activate GA biosynthesis, with GA as a negative regulator, so the net result is no effect on *VvPINs* expression. On the other hand, maybe *VvPIN4* is down-regulated by the combined IAA/GA_3_ treatment because the negative effect of GA_3_ prevails over the assumed inducing effect of IAA. In conclusion, more work needs to be done to understand the balance between the effect of IAA and GA on *VvPIN* expression.

The negative effect of GA on *VvPINs* expression was not consistent with the presence of several GA-responsive elements within *VvPINs* promoters (Additional file 3: Figure S1). However, the role of these elements is not very clear, since they are present in GA-inducible genes, but also in GA-nonresponsive genes, so the occurrence of these elements not always indicates GA responsiveness [57]. They are also present in *AtPIN1* and *AtPIN4* genes, which are repressed by GA [39].

Our results indicate that GA and IAA negatively regulate polar auxin transport, while IAA activates polar auxin transport when GA biosynthesis is inhibited. We propose that during grapevine fruitlet abscission, IAA is maintained within a high concentration range that is capable to activate GA biosynthesis, which in turn results in *VvPINs* down-regulation and hence in a reduction of polar auxin transport from 7 to 17 DAF.

As it was mentioned, the negative effect of GA on polar auxin transport was not expected since it stimulates auxin transport in Arabidopsis inflorescence stem segments [39] and in the vascular cambium of hybrid aspen [38]. However, supporting our results, it has been recently reported that GA causes an inhibition of IAA efflux in stems of hybrid aspen, affecting adventitious rooting [71]. Hence, probably GA effect on polar auxin transport is variable, and could depend on the tissue and developmental stage.

### IAA-related compounds change their content during the abscission of grapevine fruitlets

There is very few evidence regarding auxin homeostasis control in fruits. It has been reported that IAA-Asp content rises during berry ripening coinciding with IAA decrease, and thereby conjugation has been proposed to be involved in ripening initiation [49]. We found an extremely high concentration of this conjugate in grapevine fruitlets, which was in the order of micrograms per gram of tissue, while Böttcher et al. [49] reported a concentration in the order of nanograms. The reduction of this conjugate by at least 50 % from 7 to 17 DAF shows that remarkable changes in auxin homeostasis take place during abscission period (Fig. 8a). Regarding IAA oxidation, the most abundant compound was oxIAA-Glu (Fig. 8b). The content of this compound was in the order of micrograms, while IAA-Glu was in the order of nanograms, thus it seems that all IAA that is conjugated to glutamate is immediately oxidized. On the other hand, the fact that there are lower levels of oxIAA-Asp compared with IAA-Asp suggests that this conjugate is not a good substrate for oxidation. Our results suggest that IAA oxidation does not undergo strong variations from 7 to 14 DAF. Only at 17 DAF there is a significant reduction in the content of oxidized forms of IAA (Fig. 8b), possibly due to less IAA oxidation or, alternatively, to further chemical modification of these oxidized compounds. Regarding IAA biosynthesis, it seems that the regulation of IAA production is on IAM route. This compound increases significantly at 17 DAF (Fig. 8c), suggesting that inhibition of IAA biosynthesis takes place at this time. Possibly less conjugation and lower IAA export at 14 DAF compared to 7 DAF results in higher IAA content at 14 DAF (Fig. 6a), and this increase in turn inhibits IAA biosynthesis, producing an increase in IAM content at 17 DAF. The marked differences between the content of the IAA-derived compounds at 14 DAF can be observed in Fig. 8d, illustrating the preference of certain routes for IAA metabolism, being conjugation to aspartate the most prominent.

## Conclusions

**Fig. 9:**
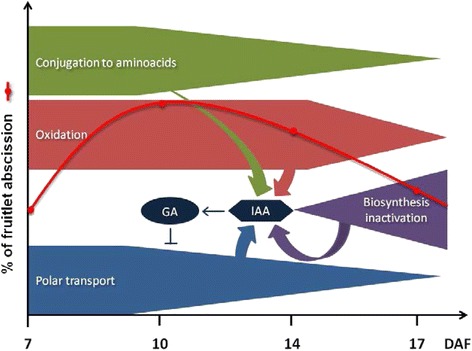
Proposed model for auxin homeostasis dynamics and polar auxin transport regulation during grapevine fruitlet abscission. Polar auxin transport and irreversible IAA conjugation to amino acids decline gradually from 7 to 17 DAF, which should result in IAA accumulation, since less IAA is exported out of the fruit or sequestered into conjugates. However, biosynthesis inactivation from 14 DAF onwards and also IAA oxidation might reduce IAA levels. As a result, IAA levels remain within a range capable to maintain GA biosynthesis activated. GA in turn inhibits polar auxin transport. As a consequence, ethylene sensitivity should be enhanced, producing an increase in fruitlet abscission

As a model of auxin homeostasis dynamics during fruitlet abscission, it is proposed that at 7 DAF high amounts of conjugated and oxidized IAA forms control IAA levels. Also polar auxin transport avoids IAA accumulation within the fruitlet. It is proposed that homeostatic mechanisms work concertedly for maintaining IAA levels within a biologically significant range, so that GA biosynthesis is maintained activated, resulting in an inhibition of polar auxin transport. Finally, the polar auxin transport decrease, with the expected increase in ethylene sensitivity, would account for abscission from 10 DAF onwards. At 17 DAF, abscission would decrease mainly due to low ethylene content (Additional file 1: Figure S2). This model is presented in Fig. 9.

In this work, GA effect on fruitlet abscission was not assessed, however it is well described the use of GA as a thinning agent when applied at the end of bloom [72]. Interestingly, after fruit set, GA does not produce berry thinning and only increases berry size [73]. Possibly, GA affects fruit retention early in fruit development, and when ethylene is low and abscission process has ended, only the well known effect of GA on berry size is observed. IAA has been also used as a thinning compound [74]. Its role in abscission would be achieved through the control of GA levels, as proposed (Fig. 9). Interestingly, treatment with 100 ppm IAA had no significant effect on grapevine fruitlet abscission, while 1000 ppm practically killed the clusters [74]. Hence, it seems that there is a range in which IAA disturbance can be buffered by homeostatic mechanisms, and out of this range the IAA effect is detrimental for normal development. It is worth to mention that in some fruit species it has been stated that IAA delays fruitlet abscission by reducing the sensitivity to ethylene, but it is important to keep in mind that this is dependent on constant auxin supply from the fruitlet to the pedicel. Here, we propose an alternative mechanism in which IAA inhibits polar auxin transport possibly through activation of GA biosynthesis. To our knowledge, this is the first time that such a mechanism is proposed.

In summary, our results show that auxin homeostasis is crucial during initial fruit development, since its disturbance via polar auxin transport inhibition leads to abscission. We proposed a model for the regulation of polar auxin transport by IAA and GA that illustrates how auxin homeostasis can be controlled. Finally, sharp variations in the content of IAA-related compounds during abscission period indicate that profound changes in auxin homeostasis occur during this period, in order to maintain optimal IAA levels. Understanding the abscission process in species such a grapevine, could contribute, in the future, to the improve agricultural practices for certain varieties and reduce fruit loss due to abscission.

## Methods

### Plant material and treatments

Three grapevine plants (*Vitisvinifera* L. cv Autumn Royal) were selected from an experimental field in the Curacaví Valley, Chile (33°36′ S, 70°39′ W) during the 2011/2012 and 2012/2013 growing seasons. In order to evaluate changes in abscission, polar auxin transport and gene expression during the abscission period, berry samples were collected at 7, 10, 14 and 17 days after flowering (DAF), with 0 DAF equal to 30 % bloom.

For assessing the effect of the inhibition of polar auxin transport on berry abscission, 10 mL of 40 μMN-1-naphthylphtalamic acid (NPA; Sigma-Aldrich) in a lanoline:vaseline (1:3) mix (NPA (+) treatment) or the mix alone (NPA(-) treatment) were applied at 10 and 20 DAF. Abscission evaluation was done 4 days post treatment (DPT). For evaluating hormonal regulation of polar auxin transport, 10 mL of 1 μM IAA (Sigma-Aldrich), 10 mL of 30 μM GA_3_ (Sigma-Aldrich), 15 mL of 1 μM IAA/30 μM GA_3_, 10 mL of 20 μM Paclobutrazol (PAC; Sigma-Aldrich) and 10 mL of 1 μM IAA-Trp (OlChemIm Ltd.) in a lanoline:vaseline (1:3) mix or the mix alone (control) were applied at 7 DAF, and the effect on polar auxin transport and gene expression was evaluated 3 DPT. To determine the role of IAA in polar auxin transport regulation in GA-deficient conditions, 10 mL of 20 μM PAC and 10 mL of 1 μM IAA/20 μM PAC in a lanoline:vaseline (1:3) mix were applied at 12 DAF and the effect on polar auxin transport was assessed 2 DPT. For each treatment the entire berry, including its pedicel, was covered with a thin layer of lanoline:vaseline (1:3) mix either alone or containing the growth regulators.

For all measurements, independent plants were considered as biological replicates, as it is shown in each figure, and samples from each one were taken between 10 am and 2 pm.

### Polar auxin transport measurements

Basipetal IAA transport assay described by Else et al. [30] was modified to measure auxin transport across excised fruitlets, through their longitudinal axis. Briefly, fruitlets were excised from the cluster under deionized water using a sharp razor blade, and a small hole was made at their apical end for placing a 0.2 μL drop of [5-^3^H]IAA (specific activity 50.55 TBq/mmol, 1 mCi/mL, 250 μCi, American Radiolabeled Chemicals Inc.), diluted 1:10 in pure ethanol (4 μM final concentration). Next, fruitlets were placed with their basal surface in contact with receiver agar discs (1.5 % (w/v) Agar-agar (Merck), 0.2 % (w/v) MES (Sigma-Aldrich), pH 5.5, in 300 μLfinal volume) arranged in a 24 well tissue culture plate (Sigma-Aldrich). After placing the [5-^3^H]IAA drop on the fruitlets, the plate was covered and kept at 22 °C, during theindicated transport periods (see legends of Figs. 1, 3 and 7). After incubation, fruitlets and agar discs were homogenized independently in 2 mL of 80 % methanol with agitation over night at 4 °C. Next, they were transferred to a vial containing 3 mL of liquid scintillation cocktail (OptiPhaseHiSafe 3, Perkin-Elmer). Radioactivity accumulated in fruitlets and agar discs was determined by radioactive scintillation counting ofdisintegrations per minute (DPM) in a liquid scintillation analyzer (Beckman Ls6500). Results were expressed as percentage (%) of polar auxin as describedpreviously [53] and values were corrected by fruitlet volume and contact surface, according to the Eq. 1:

1 $$ 100\ \left[DP{M}_{agar}/\ \left(DP{M}_{agar} + DP{M}_{fruitlets}\right)\right]\ \left(vo{l}^{1/ 3}/\ R\right)=\%\  of\  polar\  auxin\  transport $$

Where *DPM*_*agar*_is the accumulated radioactivity in the agar discs, *DPM*_*fruitlets*_is the radioactivity remaining in thefruitlets, *R* is the radius of the contact surface in the transversal cut and *vol* is the average volume estimated according to Eq. 2:

2 $$ \left[ 4/ 3\pi\ \left(T{D}^2/ 4\right)\ \left(LD/ 2\right)\right] = vol\ \left[m{m}^3\right] $$

Where *TD* and *LD* are transversal and longitudinal diameters measured using a caliper.

In the acropetal control, orientation was inverted by placing fruitlets with their apical surface in contact with the agar discs and the [5-^3^H]IAA drop was put into the fruit-pedicel junction. In NPA control, 40 μM NPA (Sigma-Aldrich) in a lanoline:vaseline (1:3) mix, was added *in planta* 24 h prior to the auxin transport experiment.

### Fruitlet abscission estimation

For abscission estimation, fruitlet number per cluster was registered by counting threads that were previously tied to the pedicels at flowering. Abscission percentage (%) was estimated according to Eq. 3:

3 $$ 100\left[ 1-\left( fruitle{t}_{final}/ fruitle{t}_{initial}\right)\right]=abscission\% $$

Where *fruitlet*_*initial*_ is the number of fruitlets registered at flowering an initial date and *fruitlet*_*final*_ is the number of fruitlets registered four days later in the same cluster. Three or four biological replicateswere performed. The values of the replicates are shown in Additional file 4: Table S1 and Additional file 5: Table S2.

### DNA sequences

CDS nucleotide sequences of *Arabidopsis thaliana* genes coding for PIN auxin transporters, *AtPIN1* [GenBank: AEE35479.1], *AtPIN2* [GenBank: AED96845.1], *AtPIN3* [GenBank: AEE35140.1], *AtPIN4* [GenBank: AEC05448.1] and *AtPIN7* [GenBank: AEE30332.1] were obtained from the GenBank database at NCBI (http://www.ncbi.nlm.nih.gov). These sequences were used as the query in a BLAT (Blast-like alignment tool) search against the *Vitisvinifera* gene predictions of the GENOSCOPE genomic database, version 12× (http://www.genoscope.cns.fr/externe/GenomeBrowser/Vitis/) to identify genes coding for putative grapevine PIN auxin transporters. Five grapevine gene models were found and named as *VvPINs* based on protein domains for each deduced amino acidic sequence, predicted using Pfam (http://pfam.xfam.org/). Three *VvPINs* were identified when *AtPIN1* sequence was used as the query, therefore they were named as *VvPIN1* (GSVIVT00017824001), *VvPIN1a* (GSVIVT00023254001) and*VvPIN1b* (GSVIVT00023255001). One *VvPIN* was found when *AtPIN3*, *AtPIN4* and *AtPIN7* sequences were used as the query, hence it was named as *VvPIN4*(GSVIVT00030482001), and one *VvPIN*was identified when *AtPIN2*sequence was used as the query, thusit was named*VvPIN2* (GSVIVT00031315001).

### Phylogenetic analysis

DeducedArabidopsis PIN protein (AtPIN) and grapevine putative PIN protein (VvPIN) amino acidic sequences were aligned using ClustalW [75]. This alignment was used to construct a phylogenetic tree in MEGA 5.05 software [76], using the Neighbor–Joining method with bootstrapping analysis (1000 replicates).

### RNA extraction and cDNA synthesis

RNA was extracted from a pool composed of seven to ten berries coming from the same cluster (0.5 g of frozen tissue) using the CTAB-Spermidine method, modified byPoupinet al.(2007) [77]. Next, RNA was treated with TURBO DNA-free™ DNase (Ambion®), following manufacturer’s instructions. RNA concentration and quality were assessed using a NanoDrop® ND-1000 spectrophotometer (Thermo Scientific™). For all samples, A260/A280 ratio values were between 1.8 and 2.0, and A260/A230 ratio values were > 2.0. For cDNA synthesis, RNA was reverse transcribed using SuperScript™ II reverse transcriptase (Invitrogen), according to the manufacturer’s instructions. Briefly, 1.5 μg of DNA-free RNA were mixed with 50 ng of random hexamers primers and 1 μl of 10 mMdNTP mix in a final volume of 12 μl. Samples were incubated at 65 °C for 5 min, and then transferred immediately to ice. Next, 4 μl of 5X First-strand buffer (Invitrogen) and 2 μl of 0.1 M DTT (Invitrogen) were added, and samples were incubated at 25 °C for 2 min. Finally, 1 μl of SuperScript™ II was added and samples were incubated for 10 min at 25 °C, 50 min at 42 °C and 15 min at 70 °C.

### PCR

PCR reactions were done in a final volume of 20 μl and Taq DNA polymerase (Invitrogen) was used. Buffers and primer concentrations (10 μM each primer) were as recommended by the supplier. PCR was conducted according to manufacturer’s instructions, under the following conditions: incubation for 3 min at 94 °C, 35 cycles of 94 °C for 30 s, 57 °C for 30 s and 72 °C for 30 s. In the final elongation step, samples were incubated for 10 min at 72 °C.

### qRT-PCR

Quantitative real-time PCR was carried out in a MX3000P detection system (Stratagene) and the SensiMix™ Plus SYBR commercial kit (Quantace) was utilized, according to the manufacturer’s instructions.

Primers suitable for amplification of 100–180 bp of *VvPIN1*, *VvPIN1a*, *VvPIN1b*, *VvPIN2* and *VvPIN4* genes were designed using Primer-BLAST tool available on NCBI webpage (http://www.ncbi.nlm.nih.gov/tools/primer-blast/). The primers are listed in Additional file 6: Table S3. In order to confirm the amplicon size and primer specificity, routine PCR reactions were made and PCR products were run on in 1.5 % (w/v) agarose gel. PCR products were excised from the gel, purified using Qiaex II (Qiagen) and sequenced. Primer efficiencies were determined by standard curves. All primers efficiencies were between 95 % and 100 %.

In order to estimate relative transcript abundance values, a ratio between the expression of the gene of interest (*GOI*) and the geometric mean of the expression of the housekeeping genes, *VvGPDH* (*VvGLYCERALDEHYDE-3-PHOSPHATE DEHYDROGENASE*, GenBank accession: *XM_002263109)* [78], and *VvUBI1*(*VvUbiquitin1*, *TC53702,* TIGR database*, VvGi5)* [79], was generated according to the Eq. 4:

4 $$ {\left(1+E\right)}^{-ct(GOI)}{\left[{\left(1+E\right)}^{-ct(VvUBI1)},{\left(1+E\right)}^{{\textstyle \mathit{\hbox{-}}}ct(VvGPDH)}\right]}^{-1/2} $$

Where E is the primer amplification efficiency value. *VvGPDH* and *VvUBI1* hadsimilar Ct values and their transcript level was stable across development and between treatments.qRT-PCR was conducted as previously reported [77], under the following conditions: denaturation at 94 °C for 2 min, 40 cycles of 94 °C for 30 s, 58 °C for 30 s, and 72 °C for 30 s.

### Immunolocalization

Sheep polyclonal anti-AtPIN4 antibody was obtained from NASC (http://arabidopsis.info/CollectionInfo?id=114). Primary antibody was diluted 1:600. AtPIN4 target sequence shares 44 % identity with grapevine homologous sequence. Sheep polyclonal anti-Human Actin-C terminal antibody (ABCAM) was used as a control. As a secondary antibody, donkey anti-sheep IgG H&L DyLight® 488 (ABCAM) was used. Secondary antibody was diluted 1:300. FM™ 4-64FX (Invitrogen) was used as a membrane stain.

For immunolocalization assays, fruitlets were fixed in 5 % glacial acetic acid, 3.7 % formaldehyde and 50 % ethanol and stored at 4 °C in the dark. Fixed samples were passed through an increasing ethanol series for complete tissue dehydration. Serial longitudinal sections of 6-8 μm thickness were cut in an HM 325 Rotary Microtome (Thermo Scientific™) and adhered to glass slides. Sections were blocked for 1 h in 1 % (w/v) bovine serum albumin (BSA) in phosphate-buffered saline (PBS) and then incubated with the primary antibody or with the preimmune serum in 1 % PBS overnight at 4 °C. Sections were washed three times in PBS, 5 min each wash. Secondary antibody was applied for 1 h in 1 % PBS in the dark. Then, sections were washed three times in PBS, 5 min each wash. FM™ 4-64FX (5 μg/ mL) was applied immediately before images were taken.

Confocal images were obtained using a Nikon Eclipse Ti C2Si microscope (Nikon Instruments Inc.). DyLight® 488 fluorescence was excited using the DPSS 488 nm laser and emission was detected between wavelengths 525 and 549 nm. FM™ 4-64FX was excited using DPSS 561 nm laser and emission was detected between wavelengths 605 and 1000 nm. Nikon Leica NIS-Elements software was used for image processing.

### LC-MS/MS analysis

For liquid chromatography-tandem mass spectrometry (LC-MS/MS) analysis, fifty milligrams of lyophilizedtissue were extracted in 3 ml of extraction solvent (methanol: formic acid: water, 15:1:4). Next, 100 μL of internal standard solution containing 20 ng of each standard was added. Extraction method is described in Gouthu et al. [80]. For each developmental stage, samples were collected by triplicate from three plants.

Standards for indole-3-acetic acid (IAA), IAA-Aspartate (IAA-Asp), IAA-Alanine (IAA-Ala), IAA-Glutamate (IAA-Glu), IAA-Tryptophan (IAA-Trp), gibberellin A1 (GA_1_), gibberellin A3 (GA_3_) and internal standards (^2^H_5_)IAA (D-IAA), (^2^H_5_)IAA-(^15^N)Aspartate (DN-IAA-Asp), (^2^H_5_)IAA-(^15^N)Glutamate (DN-IAA-Glu), (^2^H_5_)IAA-(^15^N)Tryptophan (DN-IAA-Trp), (^2^H_2_)GA_1_ (D-GA_1_) and (^2^H_2_)GA_3_ (D-GA_3_) were purchased from OlChemIm Ltd. Standards for indol-3-pyruvic acid (IPyA) and indol-3-acetamide (IAM) were purchased from Sigma-Aldrich. Standards for oxindole-3-acetic acid (OxIAA), oxIAA-Asp and oxIAA-Glu and internal standards (^2^H_2_)oxIAA (D-oxIAA) and (^2^H_2_)oxIAA-Glu (D-oxIAA-Glu) were kindly provided by Dr. HisashiMiyagawa (Division of Applied Life Sciences, Graduate School of Agriculture, Kyoto University, Japan). D-IAA was used as internal standard for IAA, IPyA and IAM. DN-IAA-Asp was used as internal standard for IAA-Asp and IAA-Ala. D-oxIAA was used as internal standard for oxIAA and oxIAA-Asp.

Hormone quantifications were done in the OSU EHSC Mass Spectrometry Facility at the Oregon State University, Corvallis, OR 97331, USA. The analyses were performed on a hybrid triple quadrupole/linear ion trap 4000 QTRAP LC-MS/MS instrument equipped with a Turbo V source (Applied Biosystems), and the analytical method used was liquid chromatography (LC)-tandem mass spectrometry in Multiple Reaction Monitoring mode (MRM) by comparison with standard curves. The transitions are reported in the Additional file 7: Table S4.

### Statistical analysis

Tukey’s media comparison analyses were performed. For all the analyses, statistical significance was assessed using *p* value < 0.05.

## Additional files

Additional file 1: Figure S2. Ethylene evolution in grapevine fruitlets. (PPTX 173 kb)
Additional file 2: Supplementary methodology. (DOCX 89 kb)
Additional file 3: Figure S1. Putative auxin and GA cis-regulators present in *VvPINs* promoters. (PPTX 147 kb)
Additional file 4: Table S1. RT-qPCR primers used in this study. (DOCX 60 kb)
Additional file 5: Table S2. Berry number per cluster for the estimation of fruitlet abscission in NPA (+) and NPA (-) treatments at 14 DAF. (DOCX 55 kb)
Additional file 6: Table S3. Berry number per cluster for the estimation of fruitlet abscission at 7, 10, 14 and 17 DAF. (DOCX 71 kb)
Additional file 7: Table S4. MRM transitions for LC-MS/MS analysis. (DOCX 66 kb)

## Abbreviations

AZ: Abscission zone
BSA: Bovine serum albumin
DAA: Days after anthesis
DAF: Days after flowering
DPM: Disintegrations per minute
DPT: Days post treatment
DW: Dry weight
GA: Gibberellin
GA_1_: Gibberellin A1
GA_3_: Gibberellin A3
IAA: Indole-3-acetic acid
IAA-Ala: IAA-Alanine
IAA-Asp: IAA-Aspartate
IAA-Glu: IAA-Glutamate
IAA-Trp: IAA-Tryptophan
IAM: Indole-3-acetamide
IPyA: Indole-3-pyruvic acid
LC-MS/MS: Liquid chromatography-tandem mass spectrometry
MRM: Multiple Reaction Monitoring mode
NPA: N-1-naphthylphtalamic acid
OxIAA: Oxindole-3-acetic acid
OxIAA-Asp: Oxindole-3-acetic acid-Aspartate
OxIAA-Glu: Oxindole-3-acetic acid-Glutamate
PAC: Paclobutrazol
PBS: Phosphate-buffered saline
*PIN*: *PIN-FORMED*
RT-qPCR: Quantitative real-time PCR
SE: Standard error
*VvGPDH*: *VvGLYCERALDEHYDE-3-PHOSPHATE DEHYDROGENASE*
*VvUBI1*: *VvUbiquitin1*
