# Supplementary material for: Regulation of polar auxin transport in grapevine fruitlets (Vitis vinifera L.) and the proposed role of auxin homeostasis during fruit abscission
Source: BMC Plant Biol. 2016 Oct 28;16:234. doi: 10.1186/s12870-016-0914-1 (PMC5084367; doi:10.1186/s12870-016-0914-1)
Supplement: Additional file 1: Figure S2. — Ethylene evolution in grapevine fruitlets. (PPTX 173 kb) [file 12870_2016_914_MOESM1_ESM.pptx]

## Slide 1
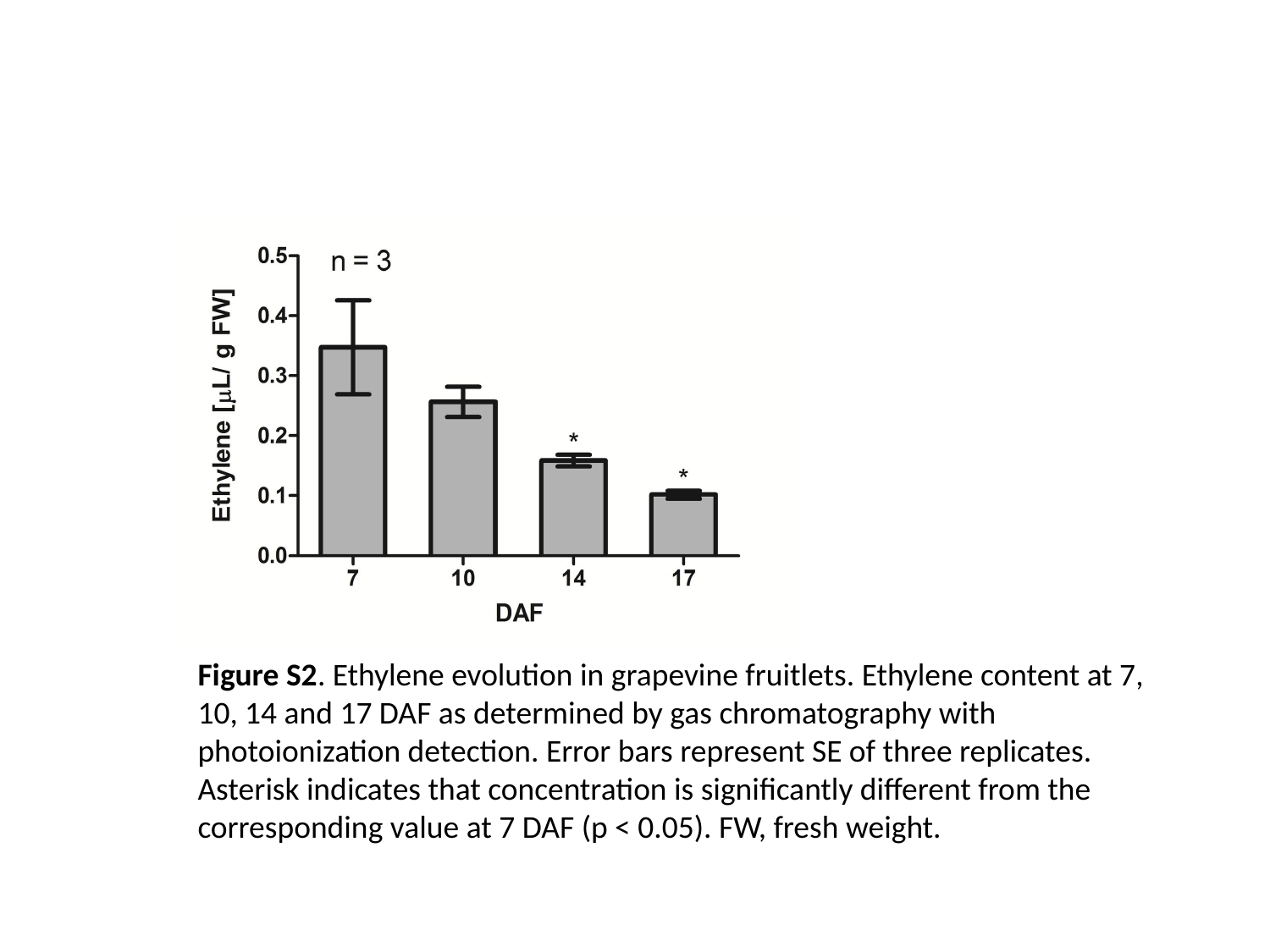

Figure S2. Ethylene evolution in grapevine fruitlets. Ethylene content at 7, 10, 14 and 17 DAF as determined by gas chromatography with photoionization detection. Error bars represent SE of three replicates. Asterisk indicates that concentration is significantly different from the corresponding value at 7 DAF (p < 0.05). FW, fresh weight.
