# Supplementary material for: Regulation of polar auxin transport in grapevine fruitlets (Vitis vinifera L.) and the proposed role of auxin homeostasis during fruit abscission
Source: BMC Plant Biol. 2016 Oct 28;16:234. doi: 10.1186/s12870-016-0914-1 (PMC5084367; doi:10.1186/s12870-016-0914-1)
Supplement: Additional file 2: — Supplementary methodology. (DOCX 89 kb) [file 12870_2016_914_MOESM2_ESM.docx]

**Supplementary methodology**

*Sequence analysis of promoter regions*

In order to detect cis-acting regulatory DNA elements, sequences of 2000 bp upstream of the grapevine and Arabidopsis genes transcription start site were downloaded from GENOSCOPE and Plant Promoter Database (PPDB) 3.0 (<http://ppdb.agr.gifu-u.ac.jp/ppdb/cgi-bin/index.cgi>), respectively. Putative cis-acting regulatory DNA elements in the promoter region were identified using PLACE database (<http://www.dna.affrc.go.jp/PLACE>).

*Ethylene determination*

Sampling of 0.8 g of berries was performed at 7, 10, 14 and 17 DAF. Berries were immediately enclosed in 2 mL tubes and maintained at 22°C for three hours and then heated at 100°C for 90 min for releasing remaining ethylene [47]. For ethylene content determination 1 mL of air sample was extracted from the headspace with a syringe and analyzed in a Photovac 10s Plus gas chromatograph (Photovac, Markham, Canada), equipped with a photoionization detector. Ethylene content in a tube with no vegetal material was measured and used for normalization. Three biological replicates were used.
