# Supplementary material for: Regulation of polar auxin transport in grapevine fruitlets (Vitis vinifera L.) and the proposed role of auxin homeostasis during fruit abscission
Source: BMC Plant Biol. 2016 Oct 28;16:234. doi: 10.1186/s12870-016-0914-1 (PMC5084367; doi:10.1186/s12870-016-0914-1)
Supplement: Additional file 3: Figure S1. — Putative auxin and GA cis-regulators present in VvPINs promoters. (PPTX 147 kb) [file 12870_2016_914_MOESM3_ESM.pptx]

## Slide 1
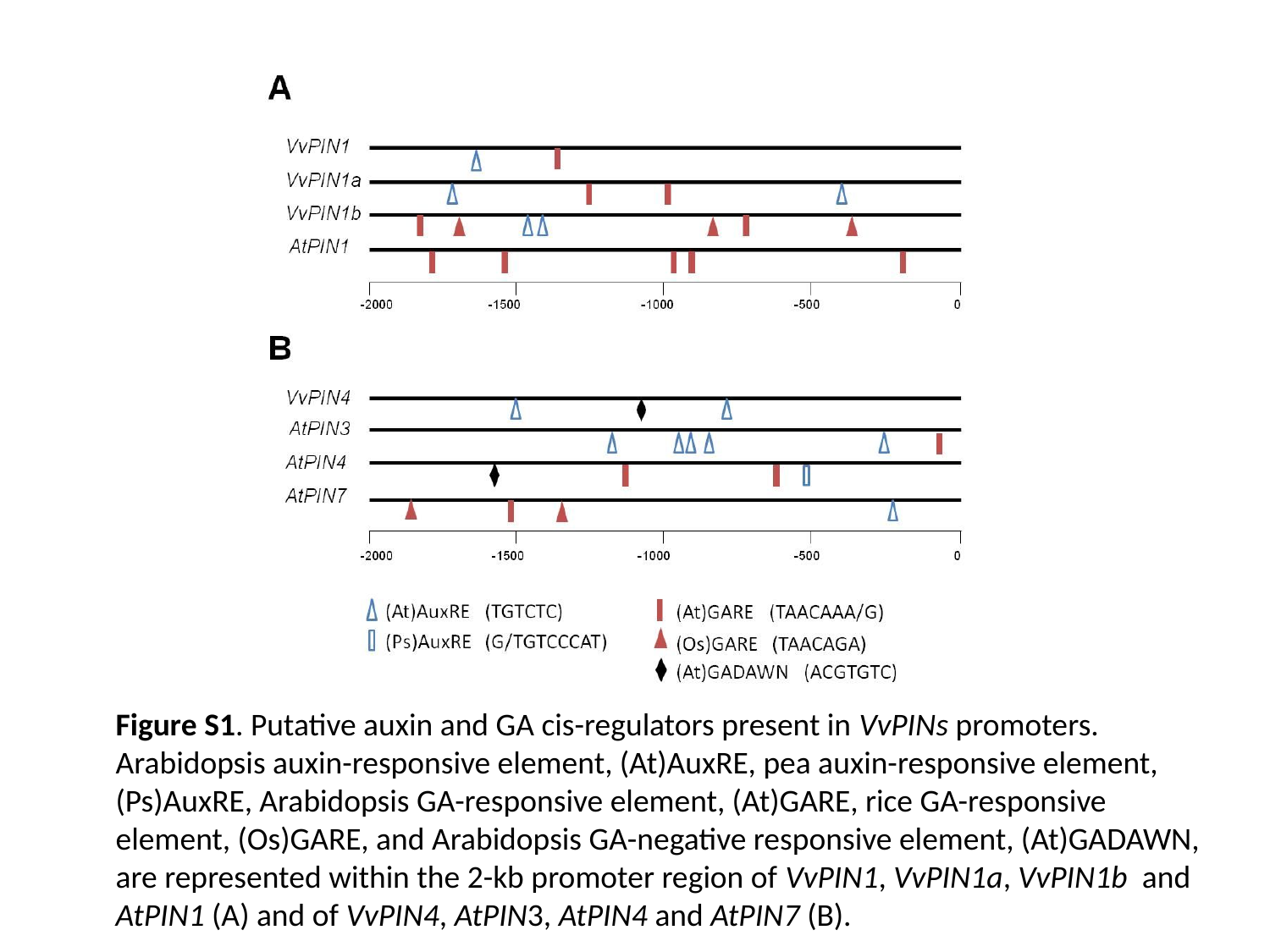

Figure S1. Putative auxin and GA cis-regulators present in VvPINs promoters. Arabidopsis auxin-responsive element, (At)AuxRE, pea auxin-responsive element, (Ps)AuxRE, Arabidopsis GA-responsive element, (At)GARE, rice GA-responsive element, (Os)GARE, and Arabidopsis GA-negative responsive element, (At)GADAWN, are represented within the 2-kb promoter region of VvPIN1, VvPIN1a, VvPIN1b and AtPIN1 (A) and of VvPIN4, AtPIN3, AtPIN4 and AtPIN7 (B).
