# Supplementary material for: Regulation of polar auxin transport in grapevine fruitlets (Vitis vinifera L.) and the proposed role of auxin homeostasis during fruit abscission
Source: BMC Plant Biol. 2016 Oct 28;16:234. doi: 10.1186/s12870-016-0914-1 (PMC5084367; doi:10.1186/s12870-016-0914-1)
Supplement: Additional file 4: Table S1. — RT-qPCR primers used in this study. (DOCX 60 kb) [file 12870_2016_914_MOESM4_ESM.docx]

**Table S1**: Nucleotide sequences of RT-qPCR primers used in this study.

| **Name** | **Primer sequences (5′–3′)** | **References** |
| --- | --- | --- |
| *VvUBI1* | F:TCTGAGGCTTCGTGGTGGTA  R:AGGCGTGCATAACATTTGCG | Downey *et al*., 2003 |
| *VvGPDH* | F:TTGGCATTGTGGAGGGTCTT  R:TTCCACCTCTCCAGTCCTTCA | Vega *et al*., 2011 |
| *VvPIN1* | F: GGTAGCGGGTGGGCGCAATT  R: ACATGCCTGGGTTGGGTGCC | This study |
| *VvPIN1a* | F: ACTCCATTTTCTCATCCGGGCGT  R: AACGACAGCCGTGAGGACGTG | This study |
| *VvPIN1b* | F: CCGCCGAGTGCCGGAATCTT  R: GACACTGGCGATGCGCTGGA | This study |
| *VvPIN2* | F: TGCGGCCATTGTGCCACTCT  R: GGAACGGCGAAAACCGCCAC | This study |
| *VvPIN4* | F: ACGCCTCGCCCCTCCAATCT  R: CGGAAACCCTGGGACCCCCA | This study |
|  |  |  |
