# Supplementary material for: Regulation of polar auxin transport in grapevine fruitlets (Vitis vinifera L.) and the proposed role of auxin homeostasis during fruit abscission
Source: BMC Plant Biol. 2016 Oct 28;16:234. doi: 10.1186/s12870-016-0914-1 (PMC5084367; doi:10.1186/s12870-016-0914-1)
Supplement: Additional file 5: Table S2. — Berry number per cluster for the estimation of fruitlet abscission in NPA (+) and NPA (-) treatments at 14 DAF. (DOCX 55 kb) [file 12870_2016_914_MOESM5_ESM.docx]

**Table S2**: Berry number per cluster at an initial date (10 DAF) and 4 days later (14 DAF) for the estimation of fruitlet abscission in NPA (+) and NPA (-) treatments at 14 DAF. Two technical repetitions (T1 and T2) for each biological replicate (R1, R2, R3 and R4) were performed.

|  | **NPA (+) replicates** | | | | | | | |  |
| --- | --- | --- | --- | --- | --- | --- | --- | --- | --- |
| **DAF** | R1 | | R2 | | R3 | | R4 | |  |
|  | T1 | T2 | T1 | T2 | T1 | T2 | T1 | T2 |  |
| **10** | 30 | 30 | 107 | 107 | 99 | 98 | 64 | 64 |  |
| **14** | 3 | 3 | 4 | 4 | 12 | 12 | 10 | 10 |  |
|  | **NPA (-) replicates** | | | | | | | |  |
|  | R1 | | R2 | | R3 | | R4 | |  |
|  | T1 | T2 | T1 | T2 | T1 | T2 | T1 | T2 |  |
| **10** | 36 | 36 | 16 | 16 | 28 | 27 | 29 | 29 |  |
| **14** | 28 | 28 | 11 | 11 | 17 | 17 | 23 | 23 |  |
