# Supplementary material for: Regulation of polar auxin transport in grapevine fruitlets (Vitis vinifera L.) and the proposed role of auxin homeostasis during fruit abscission
Source: BMC Plant Biol. 2016 Oct 28;16:234. doi: 10.1186/s12870-016-0914-1 (PMC5084367; doi:10.1186/s12870-016-0914-1)
Supplement: Additional file 6: Table S3. — Berry number per cluster for the estimation of fruitlet abscission at 7, 10, 14 and 17 DAF. (DOCX 71 kb) [file 12870_2016_914_MOESM6_ESM.docx]

**Table S3**: Berry number per cluster at an initial date (3, 6, 10 and 13 DAF) and 4 days later (7, 10, 14, 17 DAF, respectively) for the estimation of fruitlet abscission at 7, 10, 14 and 17 DAF. Three technical repetitions (T1, T2 and T3) for each biological replicate (R1, R2 and R3) were performed.

|  | **Replicates** | | | | | | | | |
| --- | --- | --- | --- | --- | --- | --- | --- | --- | --- |
| **DAF** | R1 | | | R2 | | | R3 | | |
|  | T1  87 | T2 | T3 | T1  149 | T2 | T3 | T1  137 | T2 | T3 |
| **3** | 87 | 90 | 92 | 195 | 202 | 184 | 47 | 46 | 46 |
| **7** | 85 | 84 | 85 | 160 | 162 | 166 | 45 | 44 | 44 |
| **DAF** | R1 | | | R2 | | | R3 | | |
|  | T1  87 | T2 | T3 | T1  149 | T2 | T3 | T1  137 | T2 | T3 |
| **6** | 46 | 46 | 44 | 99 | 98 | 96 | 57 | 57 | 56 |
| **10** | 19 | 21 | 20 | 40 | 42 | 41 | 36 | 36 | 36 |
| **DAF** | R1 | | | R2 | | | R3 | | |
|  | T1  87 | T2 | T3 | T1  149 | T2 | T3 | T1  137 | T2 | T3 |
| **10** | 19 | 21 | 20 | 40 | 42 | 41 | 36 | 36 | 36 |
| **14** | 13 | 14 | 14 | 36 | 36 | 36 | 29 | 29 | 29 |
| **DAF** | R1 | | | R2 | | | R3 | | |
|  | T1  87 | T2 | T3 | T1  149 | T2 | T3 | T1  137 | T2 | T3 |
| **13** | 13 | 13 | 13 | 35 | 35 | 35 | 28 | 28 | 28 |
| **17** | 13 | 12 | 12 | 33 | 33 | 33 | 21 | 21 | 21 |
