# Supplementary material for: Regulation of polar auxin transport in grapevine fruitlets (Vitis vinifera L.) and the proposed role of auxin homeostasis during fruit abscission
Source: BMC Plant Biol. 2016 Oct 28;16:234. doi: 10.1186/s12870-016-0914-1 (PMC5084367; doi:10.1186/s12870-016-0914-1)
Supplement: Additional file 7: Table S4. — MRM transitions for LC-MS/MS analysis. (DOCX 66 kb) [file 12870_2016_914_MOESM7_ESM.docx]

**Table S4**: MRM transitions for LC-MS/MS analysis. St, standard; I St, internal standard

| **Positive mode** | | |  | | | |  | |
| --- | --- | --- | --- | --- | --- | --- | --- | --- |
| **St** | | **Transition (St)** | **I St** | | **Transition (I St)** | | | |
| IAM | | 175 > 130 | D-IAA | | d5: 181 > 134 | | | |
| IPyA | | 204 > 136 | D-IAA | | d5: 181 > 134 | | | |
| IAA-Ala | | 247 > 130 | DN-IAA-Asp | | d5: 296 > 132 | | | |
| oxIAA-Asp | | 307 > 146 | oxIAA | | d2:194 > 148 | | | |
| IAA | | 176 > 130 | D-IAA | | d5: 181 > 134 | | | |
| IAA-Asp | | 291 > 130 | DN-IAA-Asp | | d5: 296 > 132 | | | |
| IAA-Trp | | 362 > 130 | DN-IAA-Trp | | d5: 367 > 132 | | | |
| IAA-Glu | | 305 > 130 | DN-IAA-Glu | | d5: 310 > 134 | | | |
| oxIAA-Glu | | 321 > 146 | D-oxIAA-Glu | | d2: 323 > 148 | | | |
| oxIAA | | 192 > 146 | D-oxIAA | | d2:194 > 148 | | | |
| **Negative** **mode** | | | |  | | | |  |
| **St** | | **Transition (St)** | **I St** | | **Transition (I St)** | | | |
| IAA | | 174 > 130 | D-IAA | | d5: 179 > 135 | | | |
| GA_3_ | | 345 > 143 | D-GA_3_ | | d2: 347 > 143 | | | |
| GA_1_ | | 347 > 185 | D-GA_1_ | | d2: 349 > 187 | | | |
|  |  | |  | | |  | | |
